# Revisiting Biological Nitrogen Fixation Dynamics in Soybeans

**DOI:** 10.3389/fpls.2021.727021

**Published:** 2021-10-07

**Authors:** Ignacio A. Ciampitti, André Froes de Borja Reis, S. Carolina Córdova, Michael J. Castellano, Sotirios V. Archontoulis, Adrian A. Correndo, Luiz Felipe Antunes De Almeida, Luiz H. Moro Rosso

**Affiliations:** ^1^Department of Agronomy, Kansas State University, Manhattan, KS, United States; ^2^Great Lakes Bioenergy Research Center, Michigan State University, East Lansing, MI, United States; ^3^W. K. Kellogg Biological Station, Michigan State University, Hickory Corners, MI, United States; ^4^Department of Plant, Soil and Microbial Sciences, Michigan State University, East Lansing, MI, United States; ^5^Department of Agronomy, Iowa State University, Ames, IA, United States

**Keywords:** soybeans, biological N fixation, soil, weather, N derived from the atmosphere

## Abstract

Biological nitrogen (N) fixation is the most relevant process in soybeans (*Glycine max* L.) to satisfy plant N demand and sustain seed protein formation. Past studies describing N fixation for field-grown soybeans mainly focused on a single point time measurement (mainly toward the end of the season) and on the partial N budget (fixed-N minus seed N removal), overlooking the seasonal pattern of this process. Therefore, this study synthesized field datasets involving multiple temporal measurements during the crop growing season to characterize N fixation dynamics using both fixed-N (kg ha^−1^) and N derived from the atmosphere [Ndfa (%)] to define: (i) time to the maximum rate of N fixation (β_2_), (ii) time to the maximum Ndfa (α_2_), and (iii) the cumulative fixed-N. The main outcomes of this study are that (1) the maximum rate of N fixation was around the beginning of pod formation (R3 stage), (2) time to the maximum Ndfa (%) was after full pod formation (R4), and (3) cumulative fixation was positively associated with the seasonal vapor-pressure deficit (VPD) and growth cycle length but negatively associated with soil clay content, and (4) time to the maximum N fixation rate (β_2_) was positively impacted by season length and negatively impacted by high temperatures during vegetative growth (but positively for VPD, during the same period). Overall, variation in the timing of the maximum rate of N fixation occurred within a much narrower range of growth stages (R3) than the timing of the maximum Ndfa (%), which varied broadly from flowering (R1) to seed filing (R5–R6) depending on the evaluated studies. From a phenotyping standpoint, N fixation determinations after the R4 growth stage would most likely permit capturing both maximum fixed-N rate and maximum Ndfa (%). Further investigations that more closely screen the interplay between N fixation with soil-plant-environment factors should be pursued.

## Introduction

Soybean (*Glycine max*. L.) is the most widely cultivated legume due to its importance as a source of protein and oil (FAOSTAT, 2015). More than three-fourth of the overall production takes place in three countries, United States, Brazil, and Argentina (USDA NASS, 2017). Despite the differences in environmental conditions among major production regions, the overall productivity of this crop relies on the biological nitrogen (N) fixation (BNF) as the most important source of N supply. As soybean yields increase over time in the last decades (Rincker et al., 2014), the need to maintain high seed protein and its energetic cost increases the reliance of plant N demand on the N fixation process. In many scenarios, increasing the overall N-gap (plant N minus fixed N) has become a growing concern at global scale (Ciampitti and Salvagiotti, 2018). Lastly, the emphasis on this N gap concept gains more relevance under the current estimates for an increase by 55% on soybean production by 2050 (Ray et al., 2013), which will outpace the rate of the expected rise in demand by ~100%.

Due to its high seed protein content, N demand in soybeans is greater relative to many other field crops (Sinclair and de Wit, 1975) and is mainly supplied by both symbiotic fixation of atmospheric dinitrogen and soil mineral N supply (Fabre and Planchon, 2000). Soybean seed yield is strongly linked to N seed uptake and the N fixation process (Ciampitti and Salvagiotti, 2018). On average, the contribution of N fixation to plant N demand ranges from 40 to 70% depending on environmental conditions (for plant growth) and the association with the host-bacteria symbiosis (Hungria and Vargas, 2000; Pauferro et al., 2010; Collino et al., 2015; Santachiara et al., 2017). Crop productivity could play a key factor in limiting the ability of the plant to sustain N fixation, with plant N requirements (80 kg N Mg^−1^) increasing with yield, mainly more prevalent when yields are above 4.5 Mg ha^−1^ (Ciampitti and Salvagiotti, 2018).

Yield limiting factors affecting plant growth such as water status, temperature, pests, and diseases will also limit the potential for N fixation (Buttery et al., 1992). Differential effects of environmental (E component) stress conditions on plant N uptake and N fixation processes have been already documented with plants more severely affected by drought (Sau and Minguez, 1990; Purcell et al., 2004; Kunert et al., 2016), flooding or oxygen stress (Minchin and Pate, 1975; Bacanamwo and Purcell, 1999; Pasley et al., 2020), light stress affecting C fixation (Schweitzer and Harper, 1980), high temperatures (Lindemann and Ham, 1979; Rawsthorne et al., 1985), and salinity (Yousef and Sprent, 1983). In addition, high soil nitrate concentration (Saito et al., 2014), low soil pH (soil acidity especially affecting the early infection process; Graham, 1992), and ineffective rhizobia (or soil rhizobia population; Thies et al., 1991) can all affect the N fixation process (Salvagiotti et al., 2008).

From a genotype × management (GxM) interaction viewpoint, the maturity group (late vs. early maturing; Patterson and LaRue, 1983), the inoculation strategy (timing, rate/dose, co-inoculation), or organic vs. conventional systems (Oberson et al., 2007; Carciochi et al., 2019) are factors to consider for identifying conditions affecting total N fixation. From a nutritional standpoint, elements such as phosphorous (Chalk, 2000) and sulfur (Divito and Sadras, 2014; Borja Reis et al., 2021) can also limit the N fixation process if they are deficient. In addition to discussing stress limiting factors affecting N fixation, the moment of occurrence, duration, and intensity of those yield-limiting stress factors need to be accounted for to fully understand the implications on plant N demand and the potential recovery for higher yield.

Seasonal N fixation is not yet well understood, with few studies investigating changes and describing evolution during the soybean development (Bethlenfalvay and Phillips, 1977; Henson and Heichel, 1984; Coale et al., 1985; Zapata et al., 1987; Herridge et al., 1990; Tamagno et al., 2018; Córdova et al., 2019). The main uncertainties linked to describing the seasonal N fixation are related to defining the overall quantity (duration by rate; N fixation, kg N ha^−1^), identifying the timing of the maximum rate of N fixation, and the proportion of the total N demand (Ndfa, %). From the Ndfa perspective, this relative proportion increases after the initial early season infection until a presumable peak. A diminishing phase is inconsistently reported after pod formation (R4) or during the seed filling period (R5–R7 stages) along with nodules senescence (Harper, 1974; Tamagno et al., 2018; Córdova et al., 2019). The time and magnitude of the peak and subsequently the declining phase are potentially linked to N limitation (for both yield and protein formation) during the critical period of seed filling (Ortez et al., 2019). In addition to describing N fixation dynamics throughout the growing season, we need to adequately acknowledge the dynamics of soil N supply (organic matter content and the mineralization process) and their influence on fixation and overall N uptake. It is well documented that soil N pools are contrariwise linked with the amount of N derived from fixation (Schipanski et al., 2010), with soil N supply reducing the amount of fixed N but without completely inhibiting this process (Allos and Bartholomew, 1955; Gelfand and Philip Robertson, 2015). Modeling the seasonal evolution of N fixation and underpinning its relationship to exogenous factors are crucial for a better understanding of processes toward designing more N-efficient farming systems.

Revisiting and providing new insights on total fixed-N in soybean, its seasonal dynamics, and the synchrony between the peaks of crop N demand and the N fixation process is critical from both productivity and sustainability viewpoints. While a few studies provided insight into when the BNF process is maximized, they were limited in terms of characterizing the seasonal variation of BNF and their association with environmental conditions. Thus, the novelty of this study is on summarizing the available in-season fixation datasets with the aims of providing insights on: (i) the time to the maximum N fixation rate, (ii) time to the maximum N derived from the atmosphere, Ndfa (%), and (iii) the importance of the main environmental factors underpinning changes over time on N fixation (kg N ha^−1^).

## Materials and Methods

### Data Collection

This database comprises temporal observations on fixed-N (kg ha^−1^) and Ndfa (%) previously published in scientific journals, in addition to two original studies. All datasets were generated from field-based experiments performed in the United States and reported at least five sampling dates throughout the soybean development cycle. Fixed-N and Ndfa were exclusively assessed through the natural abundance of ^15^N or ^15^N dilution techniques. To avoid N fixation disturbance due to N fertilization (Salvagiotti et al., 2008) and better reflect common farming practices, only treatments without (or neglectable) the application of N-fertilizer were considered. The original datasets were from field studies performed in Scandia, KS (39.7945° N, 97.7837° W) during 2019 and 2020 (Correndo et al., unpublished) and Manhattan, KS (39.1974° N, 96.5847° W) during 2019 season (Rosso et al., unpublished). Briefly, the experiments from Rosso et al. (unpublished) were set up as randomized complete block design (RCBD) with genotypes as fixed effect with the following levels: Williams 82, and three commercially available soybean maturity groups, 3.4, 3.5, and 3.7 (overall season length ranging from 115 to 120 days). All genotypes were sown on June 4. The studies from Correndo et al. (unpublished) were conducted under both irrigated and rainfed conditions with two site-years (2019 and 2020) using a soybean maturity group ranging from 3.8 to 3.9 and with the experiment arranged as an RCBD design with four replicates. The main factor of interest was N fertilizer management in the previous corn crop (control vs. N fertilized). For details on the published dataset experimental conditions, see Córdova et al. (2019) and Balboa and Ciampitti (2020).

Our analysis considered the unique combinations of year × site × sowing date and season length of each dataset, rendering 15 seasonal curves herein termed as study “id.” These combinations were chosen because they provided distinct environmental conditions. Treatments within studies that did not change environmental conditions were used as replications (*n*) for curve fitting. The most relevant characteristics of the studies evaluated (published and unpublished data) are presented in Table 1.

**Table 1:** Dataset description, study id (unique seasonal curves), author and year of publication, year of the study, number of sites, factors evaluated in the study, timing for data collection, and unique growth stages.

**id**	**Author, Year**	**Year**	**Sites**	**Evaluated factors**	**Growth stages^a^**
1	Balboa and Ciampitti, 2020	2015	1	Irrigated, dryland, row spacing, fertilizer	V4 (0.4), R1 (0.7), R3 (1.0), R5 (1.4), R7 (2)
2–3	Correndo et al., unpublished	2019 2020	1	Irrigated, dryland	V6 (0.6), V8 (0.7), R2 (0.8), R3 (1.0), R4 (1.2), R5 (1.4), R5.5 (1.7), R6 (1.8), R7 (2.0)
4–7	Rosso et al., unpublished	2019	1	Genotypes (Williams 82, P34T43R2, P35T75X, and P37T51PR)	V6 (0.6), V8 (0.7), R1 (0.7), R2 (0.8), R4 (1.2), R5 (1.4), R6 (1.8), R8 (2.5)
8–15	Córdova et al., 2019	2015 2016	2	Planting dates, environments	V2 (0.40), V3 (0.40), V4 (0.44), V5 (0.60), V6 (0.62), R1 (0.7), R2 (0.8), R3 (1.0), R3.5 (1.1), R4 (1.2), R4.5 (1.3), R5 (1.4), R6 (1.8), R6.5 (1.9), R7 (2.0), R7.5 (2.4), R8 (2.5)

a *Outside parenthesis, Fehr et al. (1971) phenology stage description. Inside parenthesis SoySim^®^ (Setiyono et al., 2010) relative phenology*.

### Phenology Scale

One of the challenges of soybean seasonal modeling is to standardize the temporal scale of crop growth in different seasons and locations. The widely used Fehr et al. (1971) phenological description is a noncontinuous scale, therefore not suitable for mathematical modeling. The soybean ontogeny is dependent on genotype and weather conditions, remarkably day length and air temperature (Egli and Bruening, 1992), which prevents the use of simple scales such as days after emergence or degree days. To standardize crop ontogeny and development across diverse conditions, phenology simulations were performed using the SoySim® (Setiyono et al., 2010). The software estimates dates of phenological stages (Fehr and Caviness, 1977), standardized from 0 (VE; emergence) to 2 (R7; beginning of physiological maturity) having 1 centered at R3 (beginning of pod formation) as a continuous dimensionless scale (Lindquist et al., 2005). Briefly, SoySim© segments phenology in (i) emergence, (ii) main stem node appearance, (iii) flowering, (iv) pod and seed set, and (v) maturity. The emergence phase is a temperature-only function. From stem node appearance until maturity, the development simulations depend on temperature and photoperiod. The temperature is based on a beta function with ideal maximum and minimum temperatures for each developmental stage. The photoperiod function is based on Yin et al. (1995), requiring a critical (*P*_c_) and an optimal (*P*_opt_) photoperiod for each phase and maturity group. If the day length is above the *P*_c_, the development rate is zero. If the day length is below *P*_opt_, the development rate is max. Day lengths between *P*_c_ and *P*_opt_ provide development rates retrieved from a photoperiod beta function. Daily radiation (MJ m^−2^), maximum and minimum temperature (°C), relative air humidity (%), rainfall (mm), and ET (mm) required by the SoySim® were acquired from the GridMet database (Abatzoglou, 2013) from sowing to harvest time at each study. Lastly, we use as an input of the SoySim© model the same maturity group of the genotype employed in each of the evaluated genotype × study combinations.

### Fixed-N and Ndfa

The fixed-N (kg ha^−1^) was estimated as the aboveground N (kg ha^−1^) multiplied by Ndfa (%) at each sampling stage. Aboveground N (kg ha^−1^) was calculated as the product of plant biomass (g), N tissue concentration (g g^−1^), and plant density (plants ha^−1^).

Nitrogen derived from the atmosphere (Ndfa, %) is a time-integrated measurement of the N percentage acquired via N fixation from the crop establishment to the sampling time. For Balboa and Ciampitti (2020), Rosso et al. (unpublished), and Correndo et al. (unpublished) datasets, the proportion of Ndfa was estimated using the natural abundance method according to the following equation (Unkovich et al., 2008), herein termed as “method 1, natural abundance”:


(1) $$\begin{array}{l} Ndfa\left(\%\right)=\frac{\delta^{15}N\;of\;reference\;plant-\delta^{15}N\;of\;soybeans}{\delta^{15}N\;of\;reference\;plant-Bvalue}\times 100 \end{array}$$

The abundance δ^15^N is the natural excess of ^15^N isotope in the soybean or reference plant aboveground biomass. The reference plant was a non-N-fixating plant, either a non-nodulating soybean plant (Balboa and Ciampitti, 2020; Rosso et al., unpublished) or maize (Correndo et al., unpublished). For Rosso et al. and Correndo et al. original datasets, it was assumed a B-value of −2.54 (median value, Balboa and Ciampitti, 2020).

Córdova et al. (2019) used the natural δ^15^N isotopic differences between nodulating and non-nodulating isolines, as well as the ^15^N dilution method, for determining Ndfa. Briefly, a month before sowing, an equivalent dose of 8.7 kg N ha^−1^ of ammonium nitrate with 99 atom%^15^N (^15^${\text{NH}}_{4}^{15}$NO_3_) was applied to the soil. The Ndfa was determined as follows (Unkovich et al., 2008), herein termed as “method 2, N dilution method”:


(2) $$\begin{array}{l} Ndfa\left(\%\right)=\\ \frac{\delta^{15}N\;of\;soybean-\delta^{15}N\;of\;atmosphere}{\delta^{15}N\;soil\;inorganic\;pool-\delta^{15}N\;of\;atmosphere}\times 100 \end{array}$$

where atom%^15^N of atmosphere is assumed as 0.3663 and the atom%^15^N of soil inorganic pool is a 3-parameter decay function of soil mineral N content at 30 cm depth (Córdova et al., 2019). In all datasets, plant tissue total N and atom%^15^N were measured by nitrogen and carbon elemental analyzer interfaced with an isotope ratio mass spectrometer.

### Environmental Covariates

The soil covariates organic matter (SOM), pH, clay, silt, and sand content from a 15-cm depth layer were acquired from POLARIS (Chaney et al., 2016); while weather covariates retrieved from GridMet were daily air temperature, air VPD, precipitation, and radiation (Abatzoglou, 2013). The candidate covariates were defined following the environmental factors associated with N fixation previously described (Borja Reis et al., 2021). Briefly, only covariates presenting Pearson's correlation |*r*| < 0.75 were kept in the environmental model (Supplementary Figure 1). Weather variables from the vegetative (V) and reproductive (R) periods were not removed even if correlated, since we want to explore their associations with the main response variables of the N fixation process. Growing season length was retained as a relevant factor since this variable combines the effect of maturity group (MG), latitude (lat), and sowing date (DOY, days of the year). Weather parameters were summarized according to the crop phenology in vegetative (V, from VE to R1) and reproductive (R, from R1 to R7) periods (Table 2).

**Table 2:** Soil (clay; soil organic matter, SOM; pH, all at 15-cm soil depth), crop/site (maturity group, season length, day of year—from January 1 to sowing time, and latitude), and weather (precipitation, radiation, temperature, and vapor-pressure deficit, VPD); covariable descriptions: mean, maximum, minimum, range, and unit of observations.

**Parameter**	**Mean**	**Min**.	**Max**.	**Unit**
Clay	30.5	23.8	34.2	%
SOM	2.4	1.7	3.1	g kg^−1^
pH	5.9	5.7	6.1	–
Maturity group	2.7	2.2	3.9	–
Season length	103	96	119	Days
Day of year	145	120	155	–
Latitude	42	39	43	Decimal degree
v^*a*^Precipitation	178	97	232	mm
vRadiation	777	437	909	MJ m^−2^
vTemperature	22.8	17.9	23.5	°*C*
vVPD	0.23	0.15	0.33	kPa
r^*b*^Precipitation	292	206	358	mm
rRadiation	1,344	1,236	1,648	MJ m^−2^
rTemperature	22.9	22.0	24.7	°*C*
rVPD	0.45	0.38	0.85	kPa

a *Observations from vegetative period (VE-R1, excluding flowering)*.

b *Observations from reproductive period (R1–R7)*.

### Statistical Analysis

Bayesian hierarchical models were implemented to describe seasonal evolution on both response variables, fixed-N and Ndfa, using the rjags package (Plummer, 2019) within the R software (R Core Team, 2020). For both response variables, two models were fit. The first model estimated an overall trend across locations, by including prior distributions connecting location behaviors (random effect). The second model was fit independently for each location, better characterizing specific behavior of fixed-N or Ndfa. In either case, 15,000 posterior samples of every parameter of interest were taken. Because Ndfa is a percentage, the first layer of the hierarchical model (data model) was defined by a parametrization of the beta distribution. The data model considers that an observation *y* at the time *i* and environment *j* (*y*_*ij*_) is generated from a beta distribution with expected value *z*_*ij*_ (true state) and a precision parameter ϕ_*j*_, given as follows:


(3) $$\begin{array}{l} \left[y_{ij}|z_{ij},\phi_{j}\right]\sim Beta\left(z_{ij}\phi_{j},\left(1-z_{ij}\right)\phi_{j}\right) \end{array}$$

The data model for fixed-N was defined as a gamma distribution with expected value *z*_*ij*_ and standard deviation sigma, as described below:


(4) $$\begin{array}{l} \left[y_{ij}|z_{ij},\sigma_{j}\right]\sim Gamma\left(\frac{{z_{ij}}^{2}}{{\sigma_{j}}^{2}},\frac{z_{ij}}{{\sigma_{j}}^{2}}\right) \end{array}$$

For the second layer of the hierarchical model (process), a deterministic nonlinear equation was adopted to describe changes on the true state (*z*_*ij*_) over the relative phenological scale (*t*). For Ndfa, the nonlinear equation was adapted from the bell-shaped function, with α_3_ multiplied by *t*, thus leading the intercept to zero. In this setting, α_1_ describes the maximum fitted value (peak), α_2_ describes the time to the peak, and α_3_ controls the rate of growth and decay (Supplementary Figure 2).


(5) $$\begin{array}{l} z_{ij}=\alpha_{1j}e^{{-\left[\frac{\left({t_{i}-\alpha }_{2j}\right)}{\left(t_{i}\alpha_{3j}\right)}\right]}^{2}} \end{array}$$

For fixed-N, the Gompertz nonlinear model was adopted to describe the process (Supplementary Table 1). Three parameters control the shape: β_1_ determines the asymptote (maximum fitted value at the plateau), β_2_ determines the moment of maximum growth rate, and β_3_ determines the maximum growth rate:


(6) $$\begin{array}{l} z_{ij}=\beta_{1j}e^{e^{-\left(t_{i}-\beta_{2j}\right)\beta_{3j}}} \end{array}$$

The priors were defined in the last layer of the hierarchical model. Biological meaning was introduced with the parameter prior distributions (Supplementary Figure 2). For Ndfa, α_1_ was described by an uninformative beta distribution, allowing the maximum NDFA to range between zero and one. The α_2_ was assumed to fall between 0.2 (early in the season) and 2 (crop maturity). The α_3_ ranged within 0.1 and 2, denoting a very abrupt to smooth decay, respectively. The precision parameter ϕ was considered to follow a uniform distribution from 1 to 300. For fixed-N, β_1_ was assumed to range from 0 to 500 kg ha^−1^, β_2_ ranged from 0.25 to 1.75, β_3_ ranged from 1 to 10, and sigma ranged from 1 to 300, all uniform prior distributions. Secondary parameters of interest, such as the total fixed-N at the end of the season, time to 50% fixed-N, and AUC, were calculated within the Bayesian framework from the parameter posterior samples.

All the posterior draws for fixed-N parameters at individual locations were linearly regressed over centered and scaled environmental covariates from Table 2, one at the time. Variable interactions with this small dataset were not pursued since parameter inference would be unlikely to improve, and the selection of those variables could result in arbitrary relationships rather than pertinent factors assisting in the study of the N fixation process. The slope distributions were recorded for each covariate and summarized by the median and the 95% credible intervals (i.e., between 2.5 and 97.5% percentiles). The distributions indicated if slopes were different from zero and the direction of the relationship.

## Results

**Figure 1:**
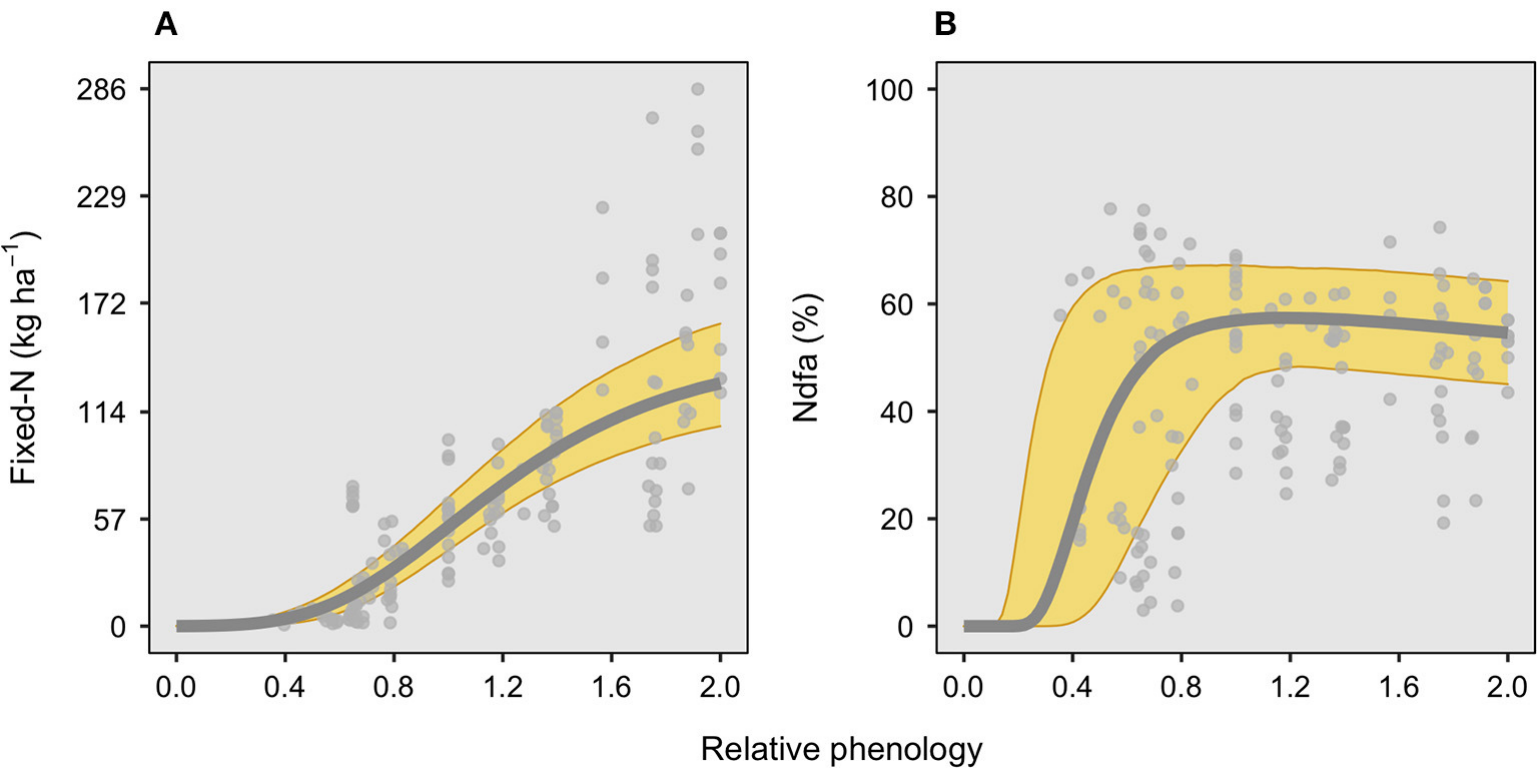
Seasonal evolution in fixed-N (kg ha^−1^) and N derived from the atmosphere, Ndfa (%), over a relative phenological scale for soybean crop (Table 1). Relative phenology scale, 0 Emergence, 1 pod formation (R3 stage), and 2 physiological maturity (R7 stage). For **(A)**, the root mean square error (RMSE) for fixed-N was 39 kg ha^−1^, while for **(B)**, the RMSE for Ndfa was 21%, with both panels comprising 15 studies with 148 observations total.

Total fixed-N (kg N ha^−1^) follows a seasonal evolution, increasing with plant ontogeny until the end of the season, when reaching an average of 130 kg N ha^−1^ (Figure 1A). Close to half of the overall fixed-N has been achieved roughly at the beginning of pod formation (R3 growth stage, 1.1, ranging from 1.0 to 1.2 in relative phenology), with great fixed-N variation during the seed filling period (R5–R7, from 1.4 to 2.0 relative phenology). For this factor, both the timing to the maximum N fixation rate and the timing to 50% to final accumulation presented a narrow variation among the studies, in relative phenology changing from 0.9 to 1.1 and from 1.0 to 1.2, respectively, for each variable. For the Ndfa (%), the time for the maximum was achieved around full pod formation (R4 growth stage, 1.15, ranging from 0.6 to 1.8 relative phenology) and remained nearly constant toward the end of the seed filling (Figure 1B). Although the maximum Ndfa (%) occurred around R4, the overall variation among studies broadly ranged from flowering to the beginning of maturity (0.6 to 1.8 in relative phenology). The time for 50% of maximum Ndfa occurred before flowering, during the late vegetative period (0.5, ranging from 0.25 to 0.75 relative phenology). The time to the maximum Ndfa occurred very rapidly, with a maximum Ndfa of 58%, ranging from 50 to 67%. Likewise, the maximum Ndfa (%), the time to half of the maximum Ndfa (%), widely varied from early vegetative to full flowering (R2) (in relative phenology ranging from 0.25 to 0.75) (Figure 1B).

**Figure 2:**
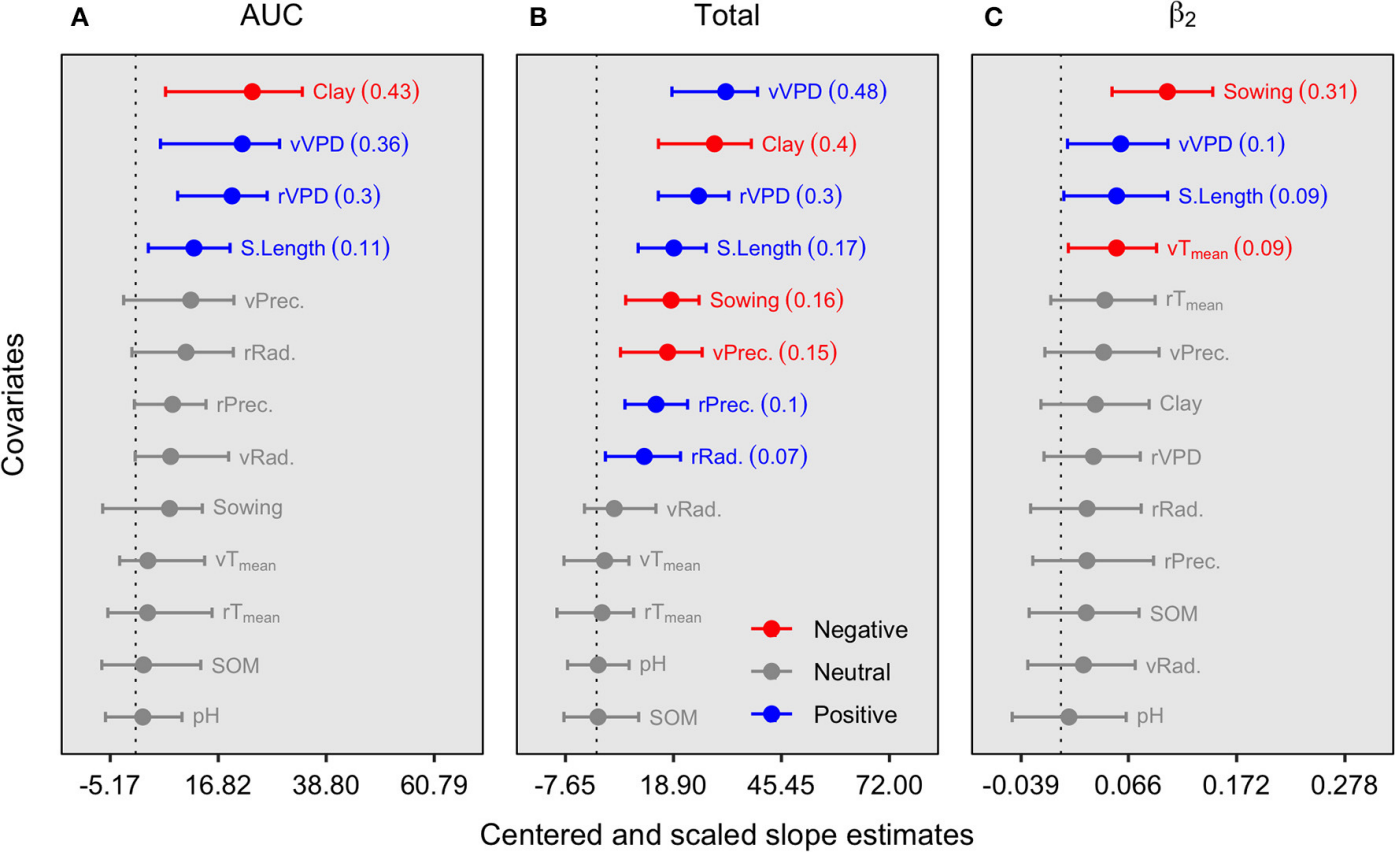
Slope magnitude and 95% credible intervals for center-scaled environmental descriptors of the AUC **(A)**, total fixed-N at maturity **(B)**, and time of maximum growth (ß2) **(C)**. The value within parentheses represents the *r*^2^ median for the respective regression. Significant slopes, either positive or negative, were determined by the 95% credible intervals. Further details on these variables and their units are presented in Table 2. Temp, temperature; tSL, total season length; Prec, precipitation; Rad, radiation; lat, latitude; DOY, day of the year; SOM, soil organic matter; r, reproductive period (R1–R7); v, vegetative period (VE-R1, excluding flowering).

To investigate the environmental association with N fixation, the AUC, total N fixation, and the time of maximum N fixation rate was reached (β_2_) were tested against environmental descriptors (Figure 2). The AUC parameter portrays the overall seasonal fixed-N, integrating all factors of the seasonal response, and depicts the seasonal strategy of N fixation. Our analysis shows that AUC was positively related to seasonal vapor-pressure deficit (VPD) and length of the growing season (S.Length) but negatively associated with the soil clay content factor (Figure 2A). The total fixed-N, peaking at the R7 growth stage (beginning of physiological maturity), was positively related to the same variables as AUC. In addition, fixed-N was also positively linked to precipitation and radiation during the reproductive period and negatively related to late planting time (sowing day of the year after January 1) and precipitation during the vegetative period (e.g., related to early-season flooding stress) (Figure 2B). The time at which the maximum N fixation rate (β_2_) was achieved was similarly affected by the environmental variables acting as significant descriptors for AUC and total N fixation, including an adverse effect of high-temperature conditions during the vegetative period, but mainly reflecting that more extended growing season (early planting and more length of the season) with suitable growing conditions is a key factor for delaying the time to the peak of N fixation rate (Figure 2C).

**Figure 3:**
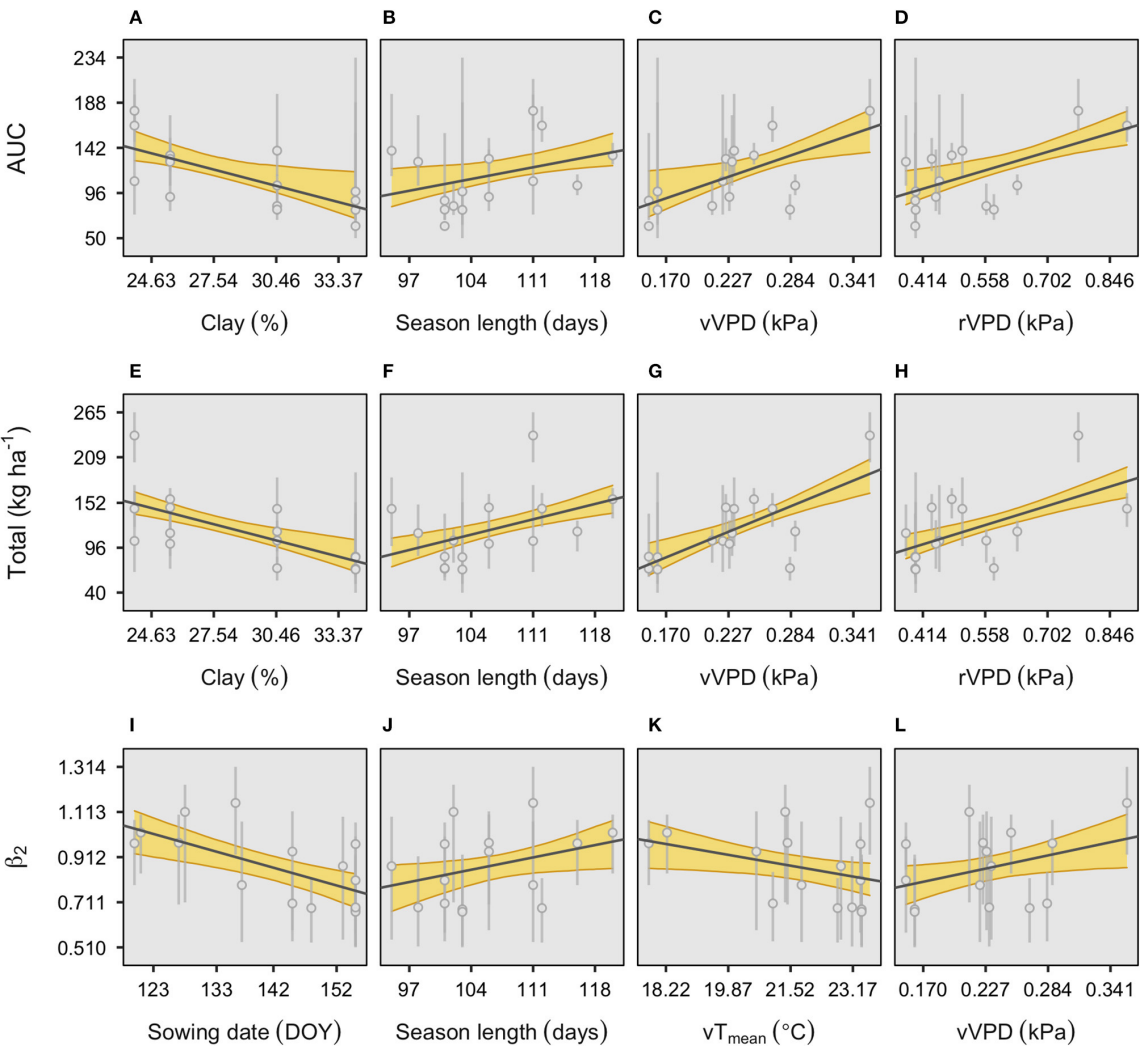
Individual plots of the four environmental effects affecting the main parameters of the fixed-N response to relative phenology, descriptors of the area under the curve **(A–D)**, total fixed-N at maturity **(E–H)**, and time to maximum growth (ß2) **(I–L)**. The shaded area represents 95% credible intervals. Error bars represent the 95% credible interval of each parameter estimate. Further details on these variables and their units are presented in Table 2. r, reproductive period (R1–R7); v, vegetative period (VE-R1, excluding flowering); VPD, vapor-pressure deficit, and *T*_mean_, mean temperature.

Individual plots for the four most relevant factors affecting the model parameters of seasonal fixed-N and relative phenology were explored to portray the direction and intensity (slope) of the trend with those environmental variables (Figure 3).

For AUC, only clay was negatively related to this factor (Figure 3A). Season length was positively associated with AUC (Figure 3B). Seasonal VPD (both vegetative and reproductive periods) presented a linear relationship with AUC, reflecting favorable growing conditions (Figures 3C,D). Likewise, similar associations with the main environmental factors (soil clay, season length, and VPD) were observed for total fixed-N (Figures 3E–H). For instance, when the clay content increases by 1%, the total fixed-N decreases on average 7 kg N ha^−1^, and when the season length increases by 1 day, the fixed-N increases on average close to 3 kg ha^−1^. Lastly, the time to the maximum N fixation rate was similarly affected by both vegetative VPD and season length as for the other two model parameters (AUC and total) but including a negative relationship (although highly variable) with vegetative temperature and with the clear effect of sowing date, with early planting with high ß2 (Figures 3I–L).

## Discussion

This study provides new insights based on the synthesis from 15 recent site-year-management combinations of seasonal variation in N fixation dynamics for soybeans. Few investigations have described seasonal patterns of N fixation (e.g., Harper, 1974; Zapata et al., 1987; Herridge et al., 1990; Herridge and Holland, 1992; Guafa et al., 1993), whereas modern research on this topic in North America has been scarce until recent years (Córdova et al., 2019; Balboa and Ciampitti, 2020). Revisiting and providing new data on seasonal (and total) fixed-N and for the peak of soybean, N demand is critical from both productivity and sustainability viewpoints. A variation on the peak for Ndfa (%) from early flowering to seed filling is expected and mainly influenced by several factors governing plant growth, soil N supply, and several other factors, including weather (temperature and water status), *Bradyrhizobium* strain, soil nutrient status, and management practices (Herridge and Holland, 1987; George et al., 1988; Hardarson et al., 1989). Although this variation is large (and expected) for the maximum level of Ndfa, the most frequent value in this study was achieved around the beginning of pod formation (R4 growth stage), as previously reported by Lawn and Brun (1974), Thibodeau and Jaworski (1975), and Imsande (1989). The latter has large implications from a phenotyping perspective; identifying the optimal timing for screening for maximum Ndfa (%) activity is a priority for the selection and breeding of more N-efficient soybean genotypes (Keyser and Li, 1992).

Unraveling the factors governing variations in the synchrony between N fixation and soil N mineralization processes still needs more attention for soybeans. From this synthesis, we evidenced that the time at which Ndfa peaks ranged from flowering to seed filling, in agreement with the reported daily rates of N fixation peaking right after flowering (R1) (Lawn and Brun, 1974; Thibodeau and Jaworski, 1975) and those studies reporting achieving a maximum during early seed filling stages (Zapata et al., 1987; Mastrodomenico and Purcell, 2012). Nonetheless, the large observed variation on maximum Ndfa reflects a lack of complete understanding of the factors controlling its seasonal variation and difficult modeling of the process itself. From a breeding perspective, the strategy of improving soybean yield is an indirect selection process for increasing overall fixed-N (Herridge and Bergensen, 1988). A study executed by Coale et al. (1985) demonstrated that improvement for yield increased plant abilities not only to fix N but also to take up the available soil N. However, the main remnant challenge is the selection for maintaining N fixation under droughty conditions (Patterson and Hudak, 1996; Sinclair, 2011).

A trade-off for yield and fixed-N under high yield could be evident under stress conditions; therefore, increasing biomass partitioning has been proposed as a plausible selection strategy for improving yields and maintaining high Ndfa (Mapope and Dakora, 2016; Tamagno et al., 2018; Córdova et al., 2019). Further investigations can focus on raising the “ceiling” for Ndfa in the US environments, with an overall total N fixation value reported here of 64%, which agrees with the country-average reported for the United States, 60% (Herridge et al., 2008). In addition, Ndfa values displayed a narrow variation range toward the end of the season, but with a more significant variation during early reproductive stages which opens the opportunity for expanding the frontier of the maximum Ndfa, but more work on improving sensitivity to soil nitrate levels among soybean genotypes (Santachiara et al., 2017) is still needed. Lastly, it is worth acknowledging that models implemented in this study are the simplification of biological processes, with an increase in model complexity limiting interpretation and practical use (Snowden et al., 2017). Future research expanding the current knowledge on seasonal fixed-N and interaction with N mineralization can explore more complex biological models to better model seasonal evolutions and for improving prediction under varying environmental factors.

This study provided more attention to weather variables favoring optimal plant growth conditions, to the length of the growing season for increasing the probability of achieving greater potential yields, and to soil variables associated with the N cycling and temporal dynamics during the crop growing season. The lack of substantial variation in soybean maturity groups (narrow pedigree) and strains (similar soil features) for the reported growing conditions should be acknowledged as potential factors critical for N fixation (Danso et al., 1987; Purcell et al., 1997; Sadras et al., 2016), needing further investigation. Seasonal VPD and season length mainly drove total fixed-N. The association with VPD is mainly governed by favorable conditions for plant growth for both vegetative and reproductive periods, regulated from the potential of biomass accumulation and N demand (Schulze, 2004; Tamagno et al., 2018; Córdova et al., 2019). The effect of season length and planting date has been reported as an effect of differences in maturity group (Tamagno et al., 2018), which changes in soybean plant biomass-to-N allocation and its potential growth (Santachiara et al., 2017). As also reported by Córdova et al. (2019), plant biomass production (reflected here as season length) was one of the best predictors of N fixation.

From a soil factor perspective, fixed-N was negatively associated with soil clay content. There are two potential hypotheses linked to this trade-off for fixed-N and soil clay content: (i) clay protection on the decay of organic matter and (ii) poor rhizosphere oxygenation due to wet conditions in high clay content soils (Schipanski et al., 2010). From the first hypothesis, although higher clay contents may relate to more soil organic matter (i.e., soil organic carbon) and the potential to provide more N, net mineralization rates tend to be reduced in soils with fine texture vs. those with coarse texture due to the capability of clay protection on the decay of the organic matter (Hassink, 1997; Castellano et al., 2012). In addition, Soinne et al. (2020) emphasized the role of clay in controlling the supply and mineralization of organic N. For the second hypothesis, the N fixation process has a larger demand on oxygen relative to root growth (Layzell and Hunt, 1990), with this process being more susceptible to poor soil oxygenation (Linn and Doran, 1984) which is exacerbated with high soil clay content (Schipanski et al., 2010). Lastly, this study expands on recent efforts on improving our understanding of the main factors underpinning that the N fixation process is still an unresolved task in our scientific community (Chalk, 2000; Hungria and Vargas, 2000; Borja Reis et al., 2021). Although the collected database is limited in size (15 studies), it is worth acknowledging that this is also a reflection of the level of efforts currently invested (at least in the last two decades) on characterizing this relevant process for soybeans in the United States. More relevant efforts should focus on collecting more detailed (with several in-season samplings) soil–plant–environmental data to better understand the N fixation process in soybeans, and this is a key research topic that warrants further attention not only from the production but from both the environmental and sustainability viewpoints for improving on our less diversified farming systems.

## Conclusion

In summary, the maximum N fixation rate was achieved around the beginning of pod formation but with the time to the maximum Ndfa attained after full pod formation. The main factors associated with total fixed-N were linked to VPD (favoring plant growth), season length (with longer duration), and clay content (likely linked to poor soil oxygenation due to wet conditions and changes in net soil N mineralization rates).

Future research should inspect more exhaustively multilayer (environmental, soil, and plant covariables) and temporal data for seasonal N fixation to build predictive models. Lastly, investigations are still needed to untangle the intricacy of co-limitations (e.g., water and N) to better understand complex interactions on plant growth and N fixation processes.

## Data Availability Statement

The raw data supporting the conclusions of this article will be made available by the authors, without undue reservation.

## Author Contributions

IC contributed to the conceptualization, methodology, original draft, funding, and project administration. AB contributed to the data curation, analysis, visualization, original draft, review, and editing. LM contributed to the methodology, data curation, analysis, visualization, original draft, review, and editing. LA contributed to the methodology, data curation, original draft, review, and editing. SA, MC, SC, and AC contributed to the data curation, methodology, review, and editing. All authors contributed to the article and approved the submitted version.

## Funding

This project was funded by support from Kansas State University provided to IC and his research Lab and contribution no. 22-042 from the Kansas Agricultural Experiment Station.

## Conflict of Interest

The authors declare that the research was conducted in the absence of any commercial or financial relationships that could be construed as a potential conflict of interest.

## Publisher's Note

All claims expressed in this article are solely those of the authors and do not necessarily represent those of their affiliated organizations, or those of the publisher, the editors and the reviewers. Any product that may be evaluated in this article, or claim that may be made by its manufacturer, is not guaranteed or endorsed by the publisher.
