# Supplementary material for: Revisiting Biological Nitrogen Fixation Dynamics in Soybeans
Source: Front Plant Sci. 2021 Oct 7;12:727021. doi: 10.3389/fpls.2021.727021 (PMC8529188; doi:10.3389/fpls.2021.727021)
Supplement: Supplementary file 1 [file Data_Sheet_1.PDF]

Supplementary Material

1 Supplementary Table

Table S1. Gompertz non-linear parameters (**β_1**, **β_2**, and **β_3**) controlling changes on fixed-N (kg ha^{-1}) throughout the soybean relative phenological scale and the standard deviation **(σ)** included for each study. Secondary parameters represent the total fixed-N at crop maturity (R7 growth stage), and area under the curve (AUC) represents the overall fixed-N as an integration of all model parameters. The root mean square error (RMSE) is presented as a measure of model fit. Posterior distribution of all model parameters is summarized by the median for each location from four datasets. Study ID represents unique combinations of geographical coordinate, genotype, or sowing date, differentiating weather and/or soil conditions.

**Database**	**ID**	**β_1**	**β_2**	**β_3**	**σ**	**Total**	**AUC**	**RMSE**
Balboa and Ciampitti (2020)	1	152	0.68	2.32	28.6	145	164	27
Correndo et al. (unpublished)	2	279	1.16	2.14	37.3	237	179	35
	3	110	0.79	2.74	48.4	105	108	41
Moro Rosso et al. (unpublished)	4	85.5	0.81	4.02	14.0	84.4	88.4	10
	5	85.8	0.68	3.86	30.4	84.1	97.7	24
	6	74.3	0.97	2.70	2.09	69.9	62.4	2
	7	71.5	0.66	3.42	27.4	69.8	79.9	21
Córdova et al. (2019)	8	174	1.02	2.33	5.76	157	134	5
	9	161	0.97	2.24	11.0	146	131	9
	10	108	0.94	2.57	12.1	101	92.2	9
	11	119	0.69	2.43	23.0	114	128	21
	12	126	0.98	2.47	6.35	116	104	5
	13	121	1.11	2.17	9.01	105	82.9	8
	14	73.6	0.70	2.48	5.78	70.5	79.3	5
	15	157	0.87	2.31	23.8	146	139	21
**Overall model**		147	1.00	2.00	18.2	129.6	113.4	39

1.1 Supplementary Figures

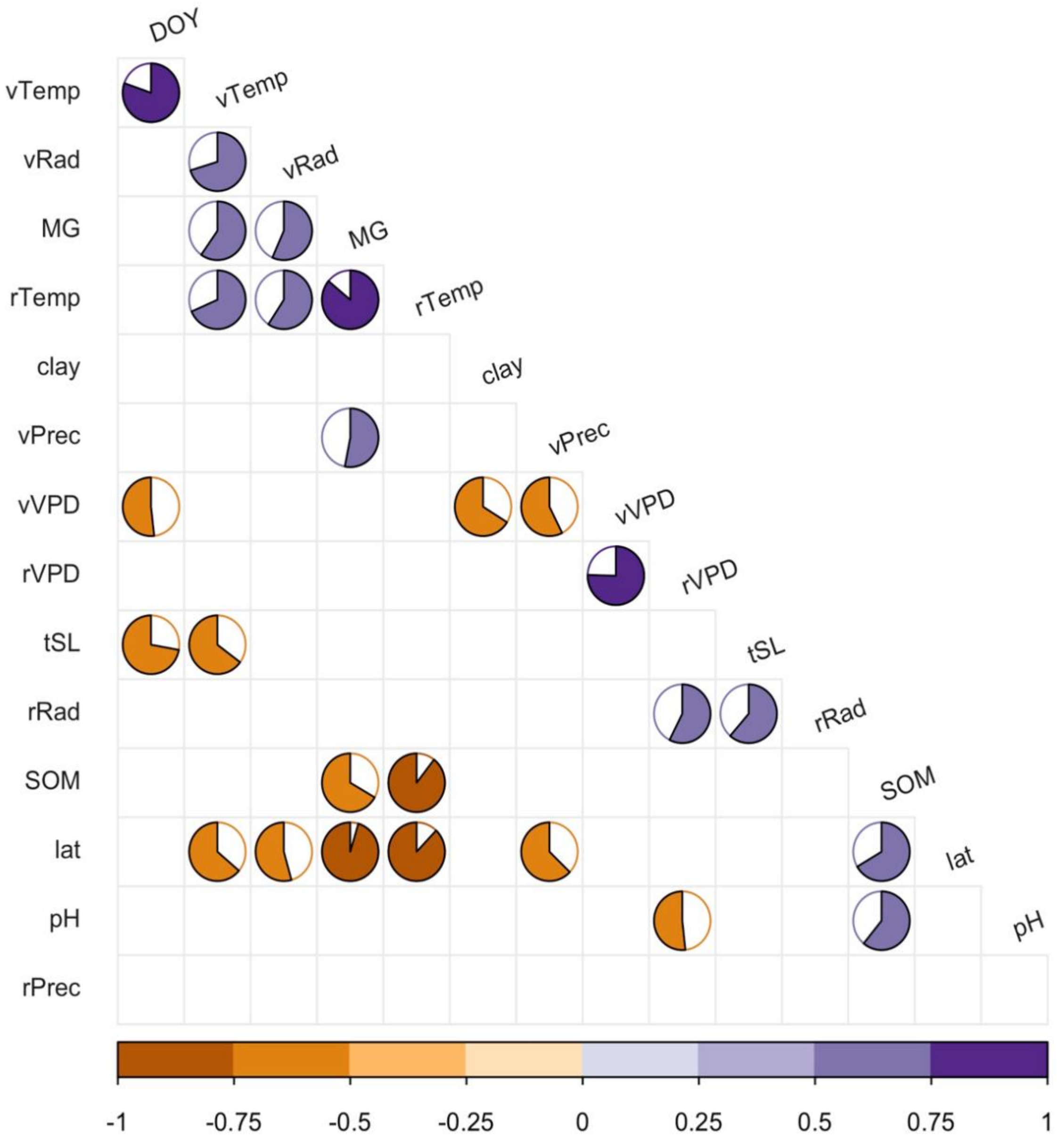

Figure S1. Correlation matrix of environmental descriptors. Significant ' 'Pearson's correlation is displayed with the pie charts, indicating the direction and magnitude of each correlation. Soil (clay; soil organic matter; pH), crop/site (maturity group, season length, day of year from January 1 to sowing time, and latitude), and weather (precipitation, radiation, temperature, and vapor-pressure deficit, VPD) variables. Further details on these variables and their units are presented in Table 2. Temp = Temperature, tSL = total Season Length, Prec = Precipitation, Rad = Radiation, lat = latitude, DOY = day of the year, SOM = soil organic matter. r = reproductive period (R1-R7), v = vegetative period (Ve-R1, excluding flowering).

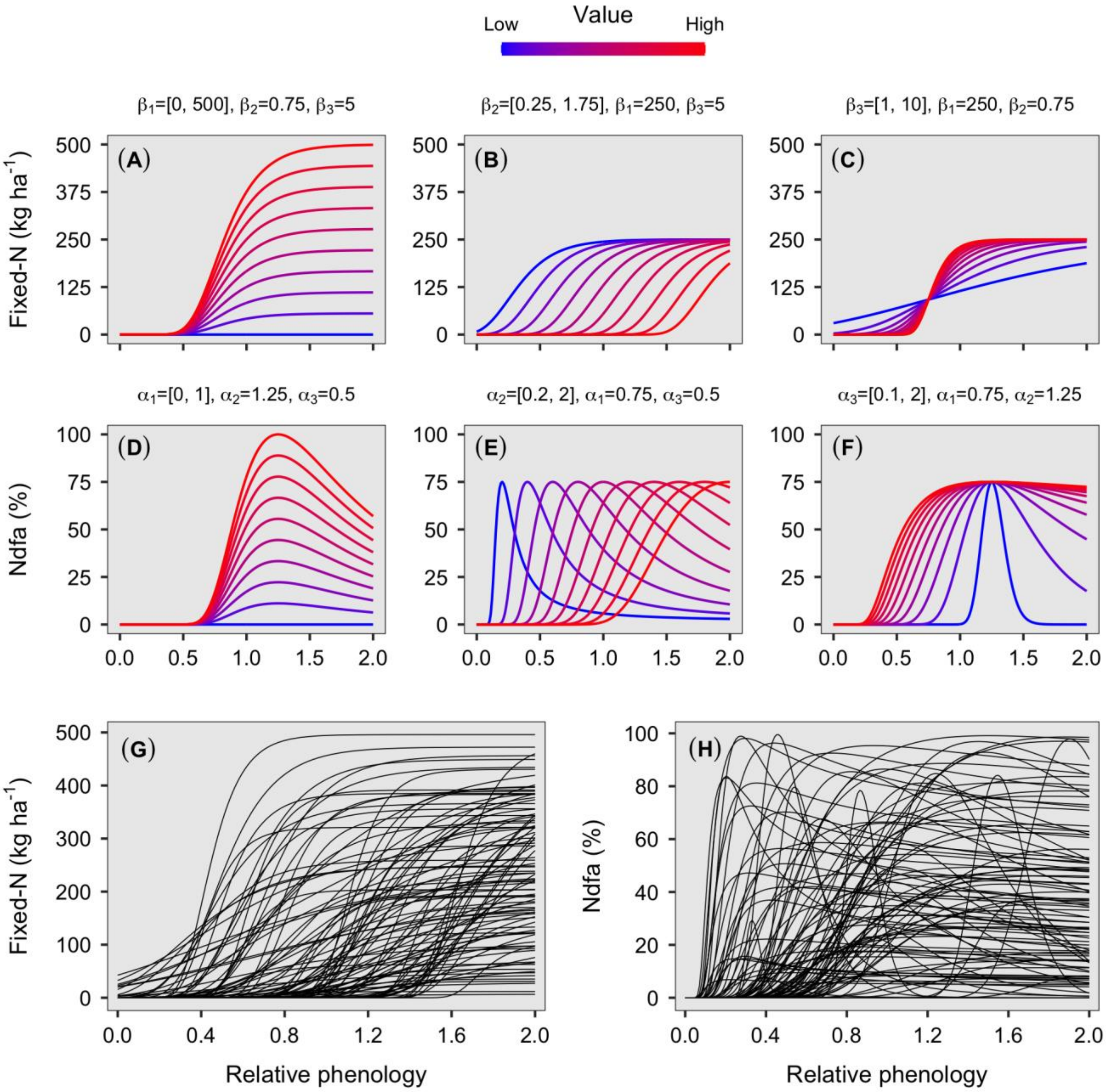

Figure S2. Independent simulations of Gompertz (a-c) and Gaussian (d-f) model parameters within the ranges of the prior distributions. For fixed-N, maximum value (B1, panel a) ranges from 0 to 500; time to maximum growth (B2, b) ranges from 0.25 to 1.75; and maximum growth rate (B3, c) ranges from 1 to 10. For Ndfa (%), maximum value ranges between zero and 100% (α1, d), time to the peak ranges from 0.2 to 2 (α2, e), and growth rate ranges from 0.1 to 2 (α3, f). The flexibility of the model priors for fixed-N (g) and Ndfa (h) is shown by 1000 independent samples of each parameter.
